# 3D optical illusion as visualisation tools in spatial planning and development

**DOI:** 10.1038/s41598-022-20173-z

**Published:** 2022-09-21

**Authors:** Rafał Kaźmierczak, Agnieszka Szczepańska

**Affiliations:** 1 Department of Spatial Analysis and Real Estate Market, Faculty of Geoengineering, Institute of Spatial Management and Geography, University of Warmia and Mazury in Olsztyn, Prawocheńskiego 15, 10-724 Olsztyn, Poland; 2 Department of Socio-Economic Geography, Faculty of Geoengineering, Institute of Spatial Management and Geography, University of Warmia and Mazury in Olsztyn, Prawocheńskiego 15, 10-724 Olsztyn, Poland

**Keywords:** Engineering, Optics and photonics

## Abstract

Spatial planning and development use various visualisation methods. Technological advancements in visualisation techniques have allowed imaging to shift from 2 to 3D dimensions. 3D optical illusion, which converts information recorded in the digital form into a three-dimensional presentation, can be a new tool for presenting spatial development solutions. Since a optical illusion is a direct spatial presentation, it requires neither specialist preparation nor spatial imagination. For this reason, it can become an effective means of visual communication with the public in the area of spatial planning and development. This article shows an example of the imaging of a model element of spatial development (a building) using the 3D illusion technique. Collected opinions of the test group of viewers confirm the usefulness of this tool. The presented 3D visualisation effect evoked positive reactions among the viewers. The use of the hologram technique in spatial planning and development appears to be justified and is an interesting research trend.

## Introduction

Design solutions for future spatial development, contained in planning documents, are presented in a graphical form along with a supplementary description. The conceptualisation of spatial development forms as spatial images is an integral part of spatial planning. The graphical form of these documents has been evolving strongly over recent years, shifting from 2 to 3D. Technological advancements in visualisation techniques have been reflected in different forms of presenting design solutions. This is very important from the point of view of both an average viewer of planning documents and the local lawmakers, who find the classic ways of presentation difficult to receive. Not only does this apply to the final version of these documents but also to their drafts made available to the public as part of the public consultation process. Specialist terminology and designations are hardly comprehensible, and the future functions and guidelines for spatial development methods are difficult to imagine. This is undoubtedly influenced by the perceptual limitations arising from both the lack of specialist training in urban planning and architecture and the underdeveloped spatial imagination. This leads to the so-called “paradox of participation”, which is defined by Ref.^1^, p. 125 as follows: “In early planning phases, when there is still sufficient room for decision-making, only a few citizens participate, while in late phases, when decisions can usually only be revised at great expense, a high level of public participation can be observed”. The reasons for this situation are attributed to the lack of clarity and the absence of concern due to a high level of abstraction.

Spatial planning is one of the areas that use the visualisation of spatial information. Difficulties with assimilating the content of planning documents and properly understanding them can affect the involvement in the public consultation process which, consequently, has an effect on the way the surrounding space is developed and the resulting spatial order, and ultimately on the quality of life. It is therefore very important to present design concepts, proposed spatial solutions and the ways of spatial development in a clear and comprehensible manner so that they can be interpreted correctly and unambiguously. The ease of reception of this information can translate into the quality of local legislation, the quality of planning documents and greater involvement in the planning process.

3D optical illusion which converts information recorded in a digital form into a three-dimensional presentation, can be a new tool for solving the problem of planning solution presentation clarity. This publication discusses and illustrates the possibilities of using this method for imaging the future state of spatial development. The article aims to show that 3D optical illusion presents spatial development elements in a manner that is clear and easy to receive directly. The model case presented in the article concerns a single-family residential building. The authors theorise that this method of visualisation of the spatial development elements allows viewers to make a quick and unambiguous interpretation, which may consequently translate into the rationalisation of future decisions. To verify this thesis, the opinions of the test group were collected to evaluate image perception in particular 3D illusion techniques. This enabled the identification of the advantages and disadvantages of the proposed tool and the most beneficial solutions from the viewer’s perspective.

## Literature review

### Spatial imagination

Spatial thinking is indispensable in everyday life because space is a fundamental category of thought^2^, and spatial thinking plays a deep role in many aspects of human cognition. Spatial knowledge, spatial perception and spatial imagination play a crucial role in solving even the most ordinary, everyday issues^3^. The ability to think in images, to perceive the visible world accurately, and to reproduce it in the mind, is called spatial intelligence (one of the seven various types of intelligence)^4–6^.

Imagination is a mental process that enables the processing of imagery. As a rule, the division of imagery refers to the senses, e.g. auditory, visual and kinesthetic imaginations are distinguished on this basis^7^. Although imagery can relate to any sensual modality, visual imagery is much more common, as sight provides up to 90% of all sensations. This is why the characterisation of imagination in terms of imaginative operation forms refers most often to visual imagination, including spatial imagination^8^.

Spatial imagination is understood as the ability to create, in the mind, an image or a geometrical object that is consistent with its actual shape and location^9^. In a broader sense, spatial imagination is defined as “the ability that combines the ability to think innovatively to find new solutions with the ability to link the existing facts and phenomena”^10^, p. 30, while in a narrower (technical) sense, as “the ability to mentally manipulate, rotate, twist, or invert pictorially presented stimulus objects”^11^, p. 893. Spatial visualisation ability is also defined as a subset of spatial ability—one of the factors of human intelligence structure^12^, p. 1. A person can be said to have spatial imagination when he/she is able, based on a picture, model, or a description, to imagine, analyse and describe the shape and location of geometric objects^9^. However, this is not an ability that everybody has. Spatial imagination is an individual characteristic determined by many factors, e.g. cognitive development, spatial experiences, gender and aptitude^12^. Researchers dealing with this issue agree on the differentiation of imaginative functions depending on gender and educational profile. According to research, men have a better developed spatial imagination than women^13^. Dependence on the educational profile is also indicated, as technical education has an advantage over education in humanities in this respect. The male model is characterised in this regard by visual and spatial abilities, good manipulation of objects in space and efficient spatial thinking^14^.

Why is spatial imagination so important? In the case of spatial planning and development, it is primarily the visual senses that are incorporated into the imaginative process, and the reception of visual stimuli results in experiencing such sensations as a colour, size, or shape^15^. Graphics is a tool used to convey engineering information, including in spatial and space-representing references. In spatial planning, information concerning a future spatial development (3D) is most often provided by means of visualisation on a plane (2D), supplemented with a description. Therefore, the viewer, when receiving and processing this information, must refer to spatial imagination. Since not everybody has this ability equally developed, only physical spatial models and modern forms of graphic presentations, including 3D illusion images, require no involvement of spatial imagination as they are themselves a spatial presentation^16^—virtual environments which are perceptually “real” even though the observer knows they are unreal^17^. The vision presented in this way is the most important for any kind of understanding of space^18^, because “..good storytelling can help to clarify the implications of different alternatives, and to build consensus by presenting particular desired or feared future developments in terms meaningful enough to be credible to non-specialists”^19^, p. 1353.

### Modern visualisation methods

It is our eyes and the brain that are responsible for spatial vision. Each eye sees images from a different angle, and the brain integrates them so that we interpret the image we see as spatial. On this basis, the cerebral cortex generates one image in three dimensions. A three-dimensional (3D) image, commonly referred to as an image with a depth, allows us to spatially locate the objects we can see. A completely different situation occurs when we are looking at a flat monitor screen, a sheet of paper, or a photograph, as our brain knows that these images are two-dimensional (2D) and flat. Solutions to allow our senses to be deceived have been developed for many years.

Based on our knowledge of the mechanics of sight, we are able to deceive the eyes and the brain, which results in our perception of flat images as three-dimensional. In order to achieve a three-dimensional effect based on a flat view, a different image must be provided to each eye. Most 3D technologies are based on this assumption. Two basic paths can be distinguished in them, namely, learning how to see spatially with the help of images generated in a special way or using specialised glasses for this purpose^20^.

Since a realistic 3D presentation is much more appealing to amateurs^21^, new solutions are being sought in this regard. Therefore, with the development of computer visualisation techniques, they are being incorporated into the process of drawing up planning documents. This has resulted in the emergence of a new form of spatial planning, referred to as e-planning using e-participation^22^. This concerns augmented reality—AR^23–31^ and virtual reality—VR^32–41^. These techniques are increasingly used in planning work to visualise the future states of spatial development. They are used to present various options of the transformations occurring in the space as part of public consultations. Another tool that can be used for this purpose is a 3D visualization colloquially called hologram^42^.

The definition of holography as a field of science and technology is widely known and most successfully reflected in the state standard of Russia^43^, which states that “holography is a branch of science and technology that studies the processes of recording, processing and reproducing information contained in in the parameters of physically realizable or mathematically described waves, using the phenomena of interference and diffraction of optical reference and object waves, as well as the possibility of practical application of these processes”. Only interference registration and diffraction recovery processes give the physical effect that Dennis Gabor called holography (full recording)^44^. The same was confirmed by the work of other founders of this science—Denisyuk^45^ and Leith and Upatnieks^46^. Lee et al.^47^, p. 10 defined a hologram as “an optical technology that records and reconstructs wavefronts of light analogously or digitally, and has exhibited potential for use in next‐generation imaging technology with various applications such as three‐dimensional holographic imaging and optical data storage”. Holographic technology allows to restore a wavefront that repeats the wavefront scattered by an object, with an accuracy of up to a wavelength, which cannot be obtained by any of the other methods of restoring 3D images. That is why holography is the basis of such a uniquely accurate measurement method as holographic interferometry. It is the ability to restore an image with an accuracy of fractions of a wavelength that is a unique property of holographic technologies, which can also be used to restore 3D images, which for the most part do not require such accuracy and can be restored by other methods.

From the point of view of these studies, the properties of the hologram are important: “holograms can reproduce very realistic three-dimensional (3D) images that satisfy all depth cues in the 3D perception of human vision without any special observation devices”^48^, p. 9087 and “holographic 3D display is thought to be the “holy grail” of 3D display technology because it is able to present truthfully a virtual window of a 3D real-world scene with all characteristics of real-world objects”^49^, p. 508. Because the hologram gives the possibility of creating the viewing environment conforming to a natural viewing condition^50^ the name hologram has been adapted to some 3D visualization optical illusion and now the “errors” in the language are embedded in society^42^. Therefore, in the common understanding of holography is best known as a method of generating three-dimensional images. Both hologram technique and 3D optical illusion (commonly known as a hologram) are applied *inter alia* in education^51–54^; medicine^55–58^, biotechnological sciences and industry^59–61^, marketing^62–65^, microscopy^66,67^, protection equipment^68,69^, and holographic cinema, television^70^.

The development of modern technologies is the result of another technological transformation. The world is shifting from an analogue economy to industry 4.0. This term describes the technological and organisational transformation of companies which, thanks to new technologies, *inter alia* digitise their products and services. The main idea behind industry 4.0 involves integrating people, information technologies and digitally controlled machines through the Internet. The ongoing changes are possible thanks to artificial intelligence algorithms, the Internet of Things (IoT), the 5G networks under construction, or, in the future, 6G networks. It is the IoT that ensures the efficient flow of information and enables further dynamic development of new technologies based, *inter alia*, in virtual reality (VR) and augmented reality (AR), which allows the definition of the Internet of Senses (IoS) to be introduced. It is full automation that allows technology to interact with other senses of sight, hearing, taste and touch^71^.

The vision of the Internet of Senses is starting to come to fruition. One of its necessary steps is the generation of digital twins. The concept of a digital twin involves creating a virtual copy of a physical object and updating it in real-time. A digital twin is an exact representation of the features and behaviour of a physical object or a process^72^. The concept of digital twins can find its application in spatial planning. In addition to virtual reality and augmented reality, it is 3D optical illusion that can also be used for the visualisation of digital spatial development models.

## Methodology

To demonstrate the possibility of using 3D optical illusion to present the future condition of spatial development elements, the visualisation of a single-family residential building was performed using a variety of 3D illusion techniques. The results of 3D visualization were presented using different techniques and 25 students were asked to evaluate them.

Below, the basic phenomena related to the principles of operation of solutions available on the advertising market under the following colloquially names hologram: holographic pyramid, polarising filters and applied LED hologram, are described. The holographic pyramid and the LED hologram are based primarily on the optical illusion of the observer.

The portion of electromagnetic radiation perceived by the retina of the human eye is commonly referred to as light, and the science that studies this issue is called optics. The concept of a light ray is a fundamental concept related to geometrical optics and is explained as a very narrow beam of light. The most important physical phenomena related to light include diffraction, fluorescence, phosphorescence, interference, luminescence, reflection, wave polarisation, rectilinear propagation of waves, light scattering, dispersion, sonoluminescence, colour temperature, stereoscopic vision, colour rendering index, refraction and the photoelectric effect. As regards the principle of optical illusion operation, the most important is the theorem of rectilinear propagation of light, which describes the general principle of the direction of light propagation, light reflection and refraction, and its polarisation^73^. The first of the above-mentioned physical phenomena is primarily used for visualisations based, for example, on special pyramids that reflect the projected image. The physical phenomenon of polarisation also finds application in the practical use of polarising filters designed to absorb polarised light. Polarising filters are most commonly used in sunglasses. As far as holograms are concerned, such filters enable stereoscopic projections. By putting on special glasses with polarising filters, an effect of spatial immersion is obtained. The final technique uses a propeller rotating faster than the human eye can see, which enables the creation of a 3D image floating in the air.

### Holographic pyramid

**Figure 1:**
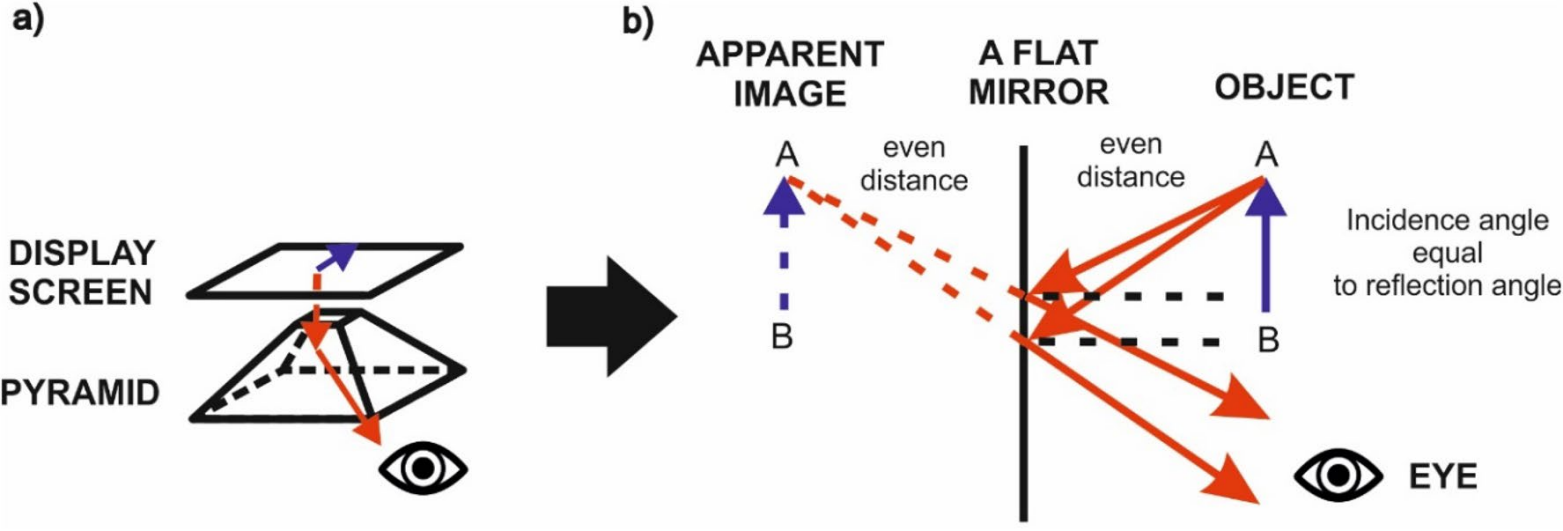
Components of a holographic pyramid (**a**) and the principle of virtual image formation in a plane mirror (**b**). *Source* own study.

The principle of displaying an object using a pyramid colloquially named holographic is very simple and based on the laws described in the previous section. The main components of the pyramid include a projection screen (which can be mounted above the pyramid) and the pyramid itself, which is constructed from a thin transparent material (preferably 1 mm thick PET plastic). A logic diagram of the principle of pyramid operation is shown in Fig. 1.

When using the laws related to the virtual image formation in a plane mirror to construct the pyramid, a virtual, simple image of the same size is obtained. This is possible due to the fact that the mirror surface is perfectly flat and the rays of light incident on it also remain parallel after reflection. A virtual image is formed when the extensions of reflected rays intersect. This is why a faithfully represented image is obtained in a mirror. This phenomenon is illustrated in Fig. 1 in part ‘b’.

When creating a holographic pyramid, it should be noted that:

- The material used to construct the pyramid should be approx. 1 mm thick, as too thick a material will result in a double view. On a thick wall, a ray of light is subject to double refraction, which results in a blurred image.
- The inclination of the pyramid walls in relation to the projection screen must be the same. Different angles can make the object being displayed smaller or larger.

### Polarising filters/3D glasses

**Figure 2:**
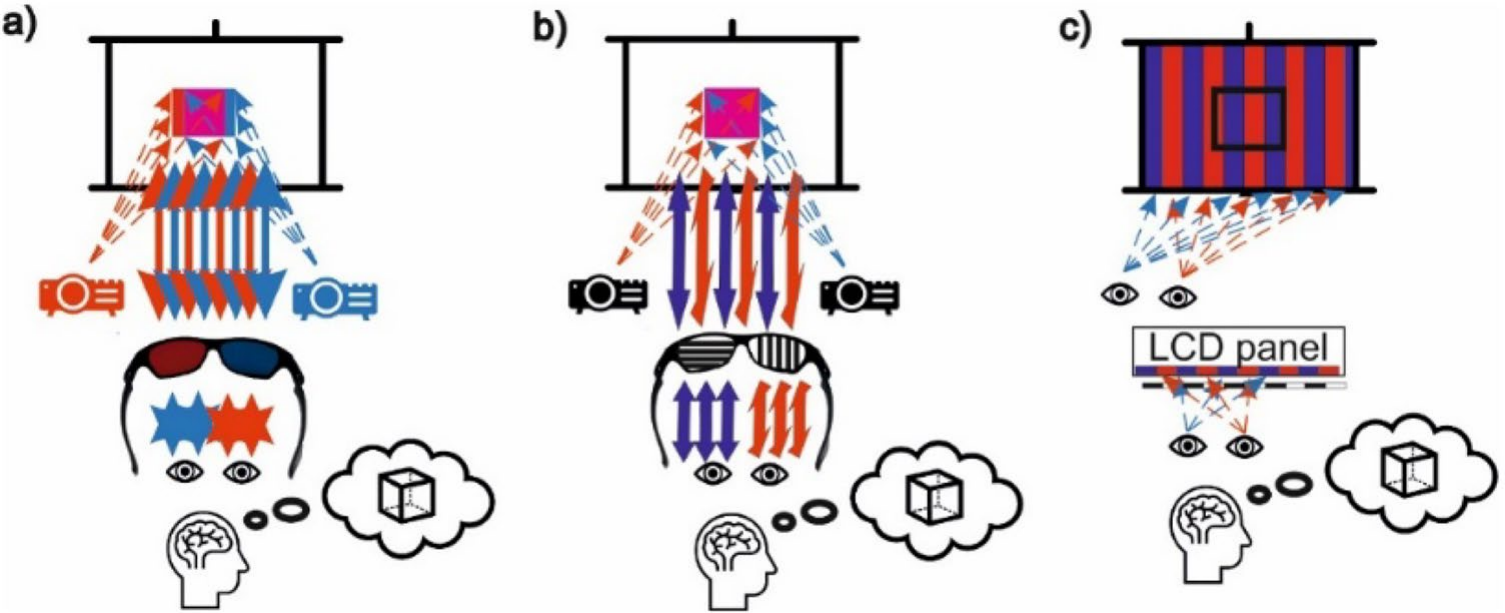
3D visualisations using the hologram technique: (**a**) anaglyph, (**b**) polarising filters, (**c**) 3D matrix. *Source* own study.

Anaglyph glasses use the oldest technique that allows a three-dimensional effect to be obtained. In this technique, a different image is delivered to each eye. Two superimposed images in two different colours (red and blue), slightly offset from each other, are generated on the monitor screen (Fig. 2a). The glasses have appropriately coloured lenses, one is red and the other blue. Each lens of the glasses blocks out blue or red light rays, and, thanks to this procedure, after putting the glasses on, each eye sees a different photograph, which gives an impression of depth. Without the anaglyph glasses put on, an anaglyph photograph looks like a blurred image. The limitation of this technique is the limited colour scheme^74,75^.

Another example of the glasses are passive 3D glasses. The principle of their operation is based on polarisation stereoscopy. In this case, two different images are delivered to the eyes as well but using a completely different method. In order to generate the three-dimensional effect, special monitors or two still projectors with polarising filters with the light polarisation directions perpendicular to each other are used (Fig. 2b). As a result, two horizontally positioned lines are polarised clockwise and anti-clockwise. The user’s glasses also have polarising filters installed, so that the eye receives images of the same resolution, offset from each other. A specially adjusted image is delivered to each eye. Using this method, a high-quality image that can also be viewed at different angles is obtained^76^.

A third example of 3D glasses are shutter glasses, also known as active glasses. In order to achieve the depth effect, it is necessary to synchronise liquid crystal shutter glasses, a powerful graphics card in a computer and a monitor with a high refresh rate (at least 120 Hz). The principle of operation involves displaying two different images alternately on the monitor screen at a very high frequency (120 times per second). The use of active glasses allows these images to be extracted and delivered individually to each eye. Synchronisation of the glasses with the display allows the left and right eye to be alternately dimmed so that the observer believes that he/she can see the image in 3D (Fig. 2c). This method produces a very realistic image with no colour limitations.

### 3D matrices

One of the possibilities for spatial vision is also the use of special matrices that enable 3D vision without the need to wear special glasses. In this case, it is the monitor screen that displays a different image to each eye. These are usually autostereoscopic 3D TV sets, either equipped with two LCD matrices or displaying half the pixels for one eye and the other half the pixels for another eye. This is a complex technology whose main limitation is the small angle of view, and the required short distance between the observer and the monitor^77,78^.

### LED hologram

**Figure 3:**
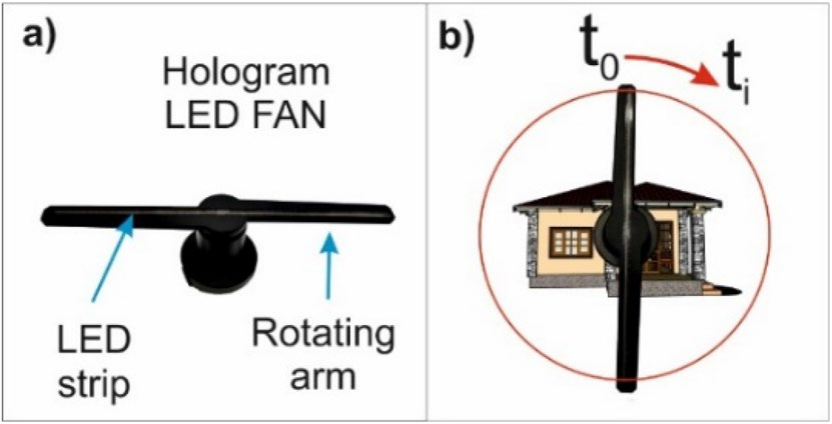
Diagram of LED hologram operation: (**a**) LED hologram elements, (**b**) general operation diagram. *Source* own study.

The principle of LED hologram operation is based on a rotating arm on which an LED strip is placed. Due to the rapid rotation of the arm (Fig. 3a), the human eye can see a complete object being displayed (Fig. 3b). Before displaying the object (or an animation with the object), the area of the film being displayed, which coincides with the LED strip length, needs to be defined. A part of the image or film being displayed is assigned to individual LEDs. A necessary factor for obtaining a 3D effect is the need for the object being displayed to move. A limitation of this display method is the correlation of the rotating arm and the positions of individual diodes with the animation being displayed. Another major limitation is the range of colours that can be displayed, which corresponds to the LED strip colours. In the final outcome, a streaky effect caused by the rotation of the hologram arm can be observed.

### Statement

Authors confirm that informed consent was obtained from all survey studies participants. Survey studies were carried out in accordance with relevant guidelines and regulations, in an anonymous manner such that the information cannot be traced back to the individual user. The survey studies were approved by Research Ethics Committee.

## Results

When using the 3D visualisation technique described above, a single-family house, i.e. an element of spatial development, was imaged. The first imaging used the holographic pyramid technique. In order to display a 3D image, a visualisation of a single-family house was prepared in the first stage. The visualisation was a film showing the object from the bird’s-eye perspective, with smooth transitions, rotations and different view perspectives. In order to display the object correctly, its proportions on the projection screen needed to be correctly selected. It should also be remembered that only the object itself should be displayed, preferably against a black or very dark background. The removal of background (e.g. the sky) better reveals the advantages of the presented object while enhancing the obtained depth effect.

**Figure 4:**
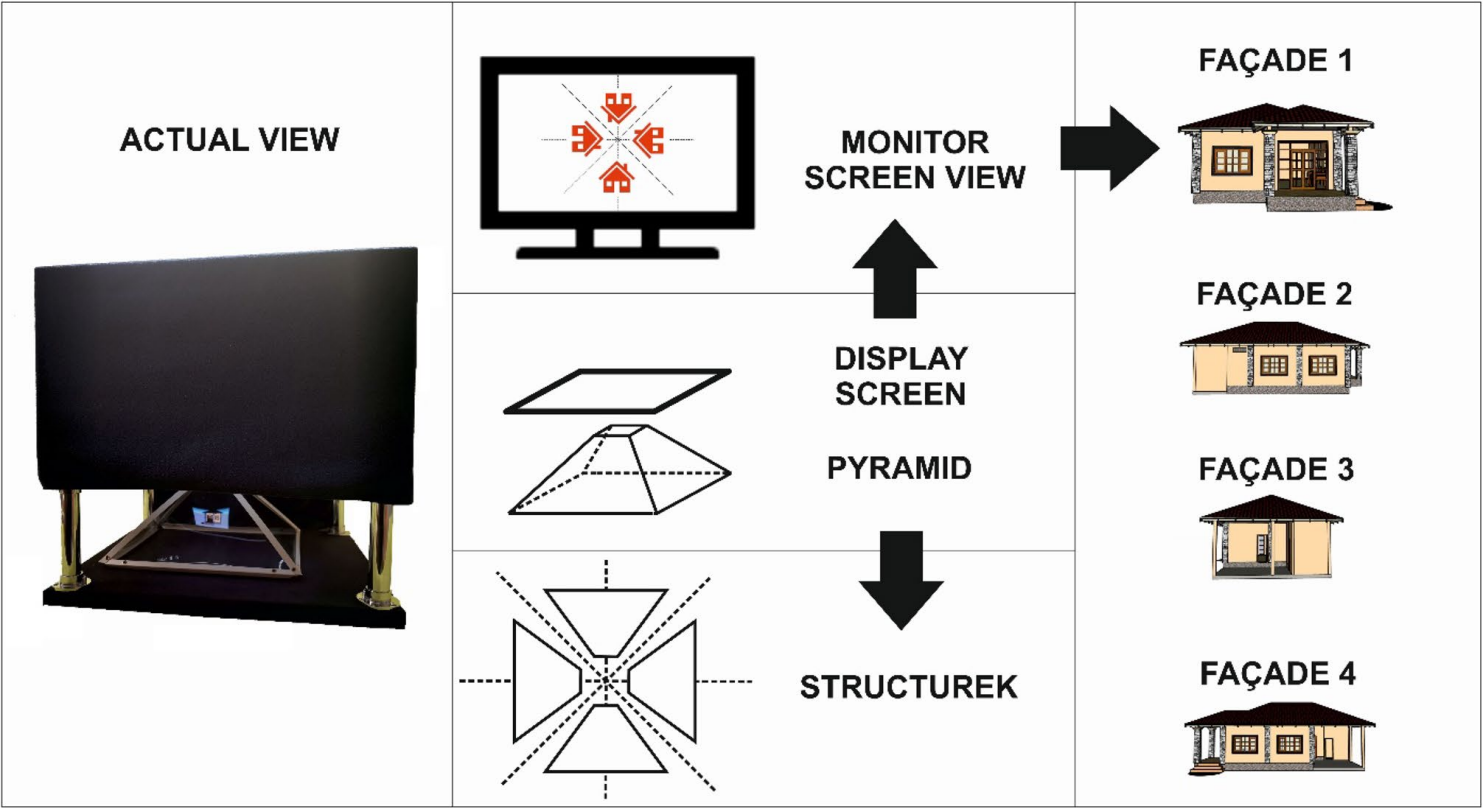
Visualisation of a single-family house using a 3D holographic pyramid. *Source* own study.

The animation prepared in this way was displayed in four planes pointing with the top towards the centre of the monitor, which coincided with the pyramid top. Due to such a visualisation, the wall of the house was visible on each of the four pyramid walls. In this case, the mutual synchronisation of the four walls being displayed was very important, as it allowed the observer, moving around the platform with the holographic pyramid, to smoothly observe the object being shown. The results of the holographic pyramid use are provided in Fig. 4.

**Figure 5:**
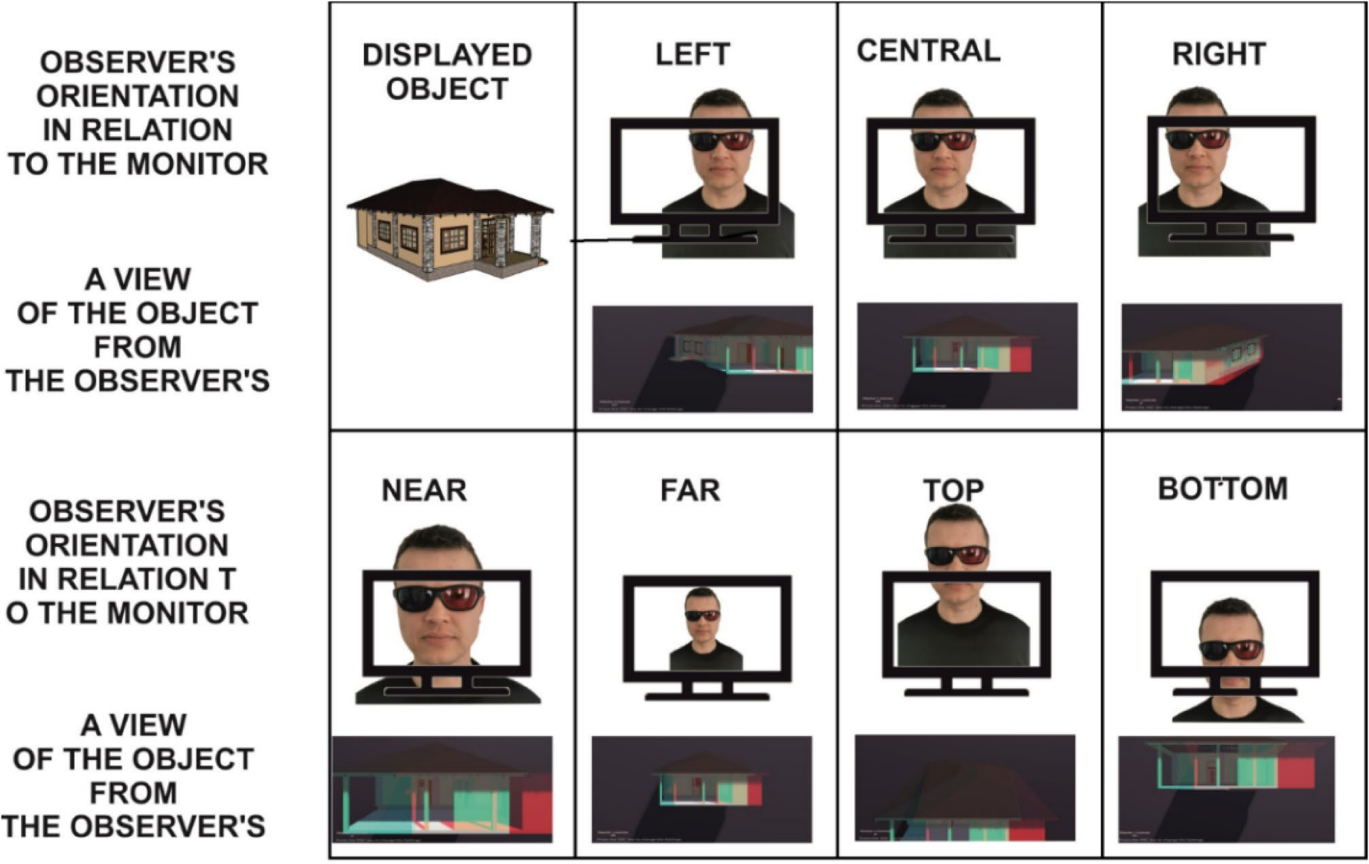
Diagram of Holo-SDK operation from the observer’s perspective. *Source* own study.

Another imaging of the same single-family house was prepared using the technology applying 3D glasses. To this end, the Holo-SDK environment was used (https://www.holo-sdk.com/—access on 26-10-2021). This is a holographic software development kit (SDK) which is compatible with every laptop and PC. The main idea behind the SDK is to display a hologram based on the tools and algorithms that track the viewer’s head position. In this way, the generated image is personalised and determined by the observer’s head position. This environment enables the rendering of objects as red-and-blue images for the Anaglyph 3D mode or in the side-by-side format for both passive and active 3D displays. The Head-tracking 3D function enables real-time tracking of the user’s head position and rendering of scenes depending on the viewing angle. The observer obtains a 3D effect, and the image changes its positions during the observation. The final effect of the operation of this environment is shown in Fig. 5. This solution uses separate colour coding for the left and the right eye. Combinations of blue and red are the most common for the anaglyph glasses, and these particular glasses were used. An image is displayed for the observer on a standard monitor, and after putting on the glasses, he/she obtains (depending on the position of the head) a 3D effect of the object.

Figure 5 shows the effect of head position in relation to the monitor and the resulting view. Leaning the head in any direction makes the object lean in the opposite direction. Hence, we obtain the effect of looking into what is happening on the side of the object while at the same time looking at the object as if it were a 3D spatial object. In addition to using the Anaglyph 3D visualisations, the Holo-SDK environment enables the use of both active and passing 3D solutions. In order to operate, these solutions require specialised monitors and glasses, which allows the observer’s sensations to be changed.

**Figure 6:**
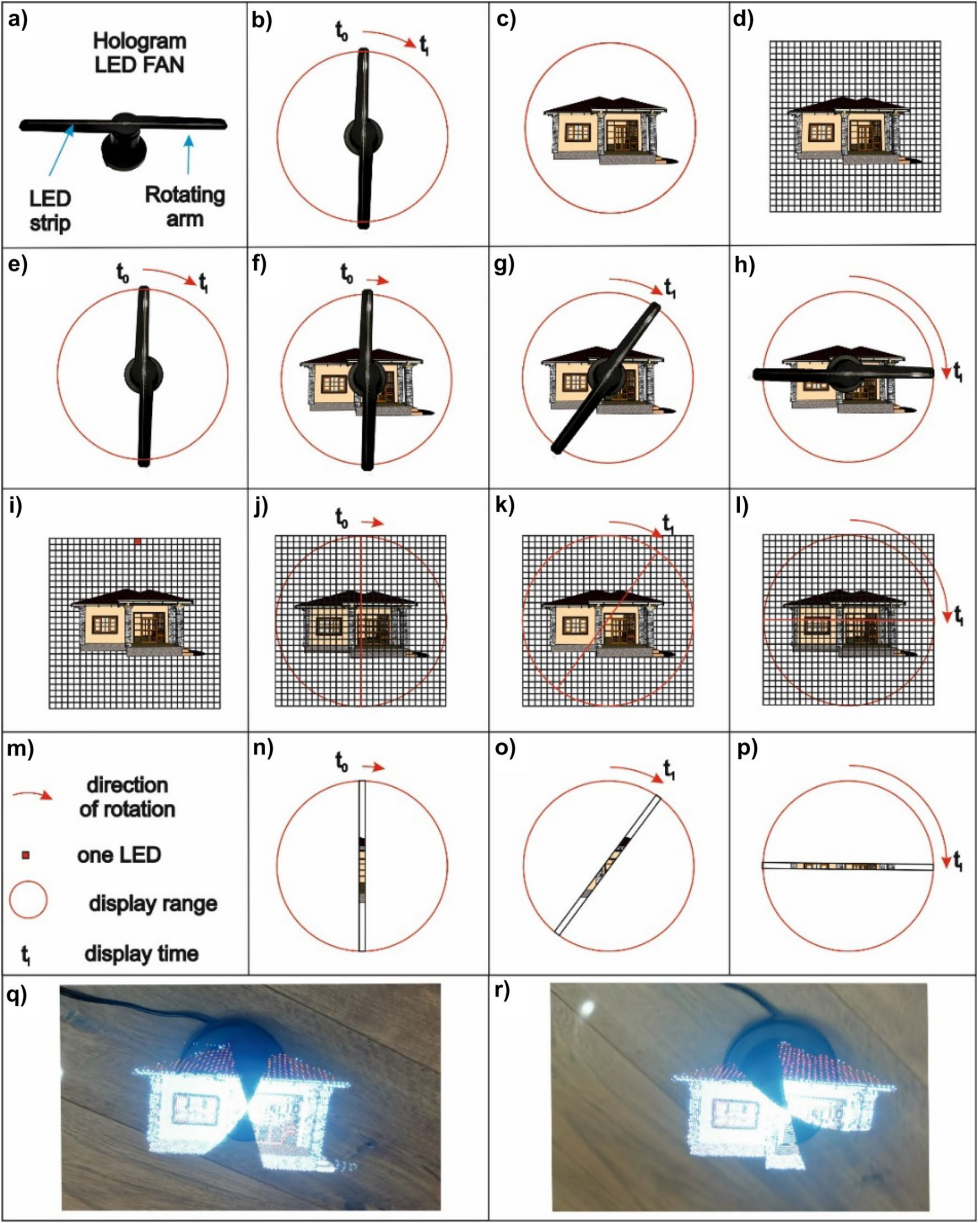
Visualisation of a single-family house in an LED FAN hologram: (**a**) LED FAN hologram; (**b**) direction of the arm’s rotation; (**c**) object display area; (**d**) the object being displayed with a network of squares; (**e–h**) animation of movement depending on the observation epoch; (**i–l**) object display logic; (**m**) legend; (**n–p**) single LEDs with different colours displayed; (**q,r**) different visualisation epochs. *Source* own study.

For the third visualisation of the building, an LED hologram was used. As previously mentioned, the operating principle of the device is based on a rotating arm on which an LED strip is placed (Fig. 6a,b). Due to the rapid rotation of the arm (Fig. 6e–h), the human eye can see a complete object being displayed (Fig. 6d). Before displaying an object or an animation with an object, the area of the film being displayed (which coincided with the LED strip length) was defined (Fig. 6c). During one epoch (t_0_) of image or film display, one square from the image on the photograph (Fig. 6d) is assigned to each single LED (Fig. 6i). In this way, knowing the location of the arm and the location of a single LED in relation to the image, the appropriate display colour can be assigned to the set time (ti) (Fig. 6j–l). The essence of clearly displaying an object is to properly prepare the input file for display. It is important to note that the object should be placed in the centre and that the background of the object should remain black. Adding motion animation, i.e. rotation and zooming, produces a spatial effect. The observer sees the object located in the air, and its motion creates a 3D effect. When an object animation is displayed on single LEDs, the assigned view from the animation will change depending on the moment of animation (Fig. 6m–p). Figure 6q,r show two photographs for different display times. The photograph captures and shows the location of the arm’s movement, but when the image is perceived by the organ of sight, the image is virtually complete.

## Discussion and conclusions

**Figure 7:**
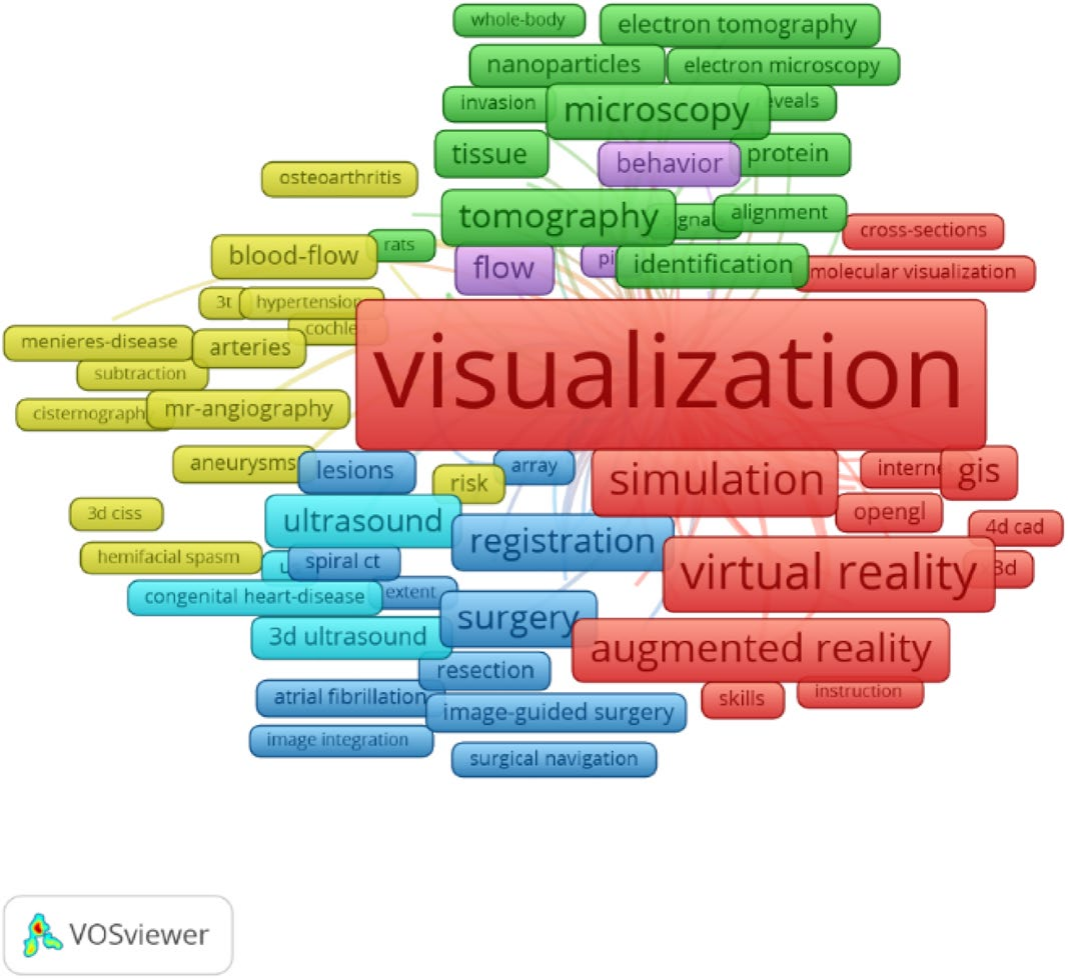
Analysis of keywords using the VOSviewer program (Holo-SDK 1.0 in Unity, https://www.holo-sdk.com/) for the keyword “Visualisation 3D”. *Source* own study.

In order to examine the trends related to the possibilities of presenting planned spatial solutions using three-dimensional visualisation, the Web of Science database was analysed with respect to two keywords: Visualisation 3D and Hologram 3D. In both cases, studies from the period of 1900–2021 were considered. When carrying out the literature review, the authors followed the “from the general to the specific” principle. For the term “Visualisation 3D”, there were approx. 26,000 studies in the database, which contained 62,256 keywords, of which 4744 keywords were accepted for the visualisation presented below (only those that appeared at least five times were left). Figure 7 shows the analysis of keywords in filtered studies for the keyword “Visualisation 3D”.

The overall analysis shows a strong link between 3D visualisation and simulation, virtual reality (VR), and augmented reality (AR). In this area, the most frequently appearing keyword according to the “Total link strength” criterion (total link strength attributes indicate, respectively, the number of links on an item with other items, and the total strength of the links of an item with other items) was visualisation (Table 1).

**Table 1:** The result of a keyword search for “Visualisation 3D”.

Keyword	Occurrences	Total link strength
Visualisation	4481	14,767
Reconstruction	821	3430
3D	791	3101
System	683	2800
Model	624	2360
3D visualisation	1070	2345
Segmentation	550	2245
Virtual reality	878	2202
CT	457	2164
Simulation	652	2075

*Source* own study.

In the presented figure, the size of the label shows the greater weight of a keyword in the analysed publications. The colour of the element is determined by the cluster. The lines between labels correspond to the network of relationships, and the distances between the labels inform about the relatedness of the concepts. Detailed instructions for graphical interpretation are provided in the VOSviewer Manual^79^.

**Figure 8:**
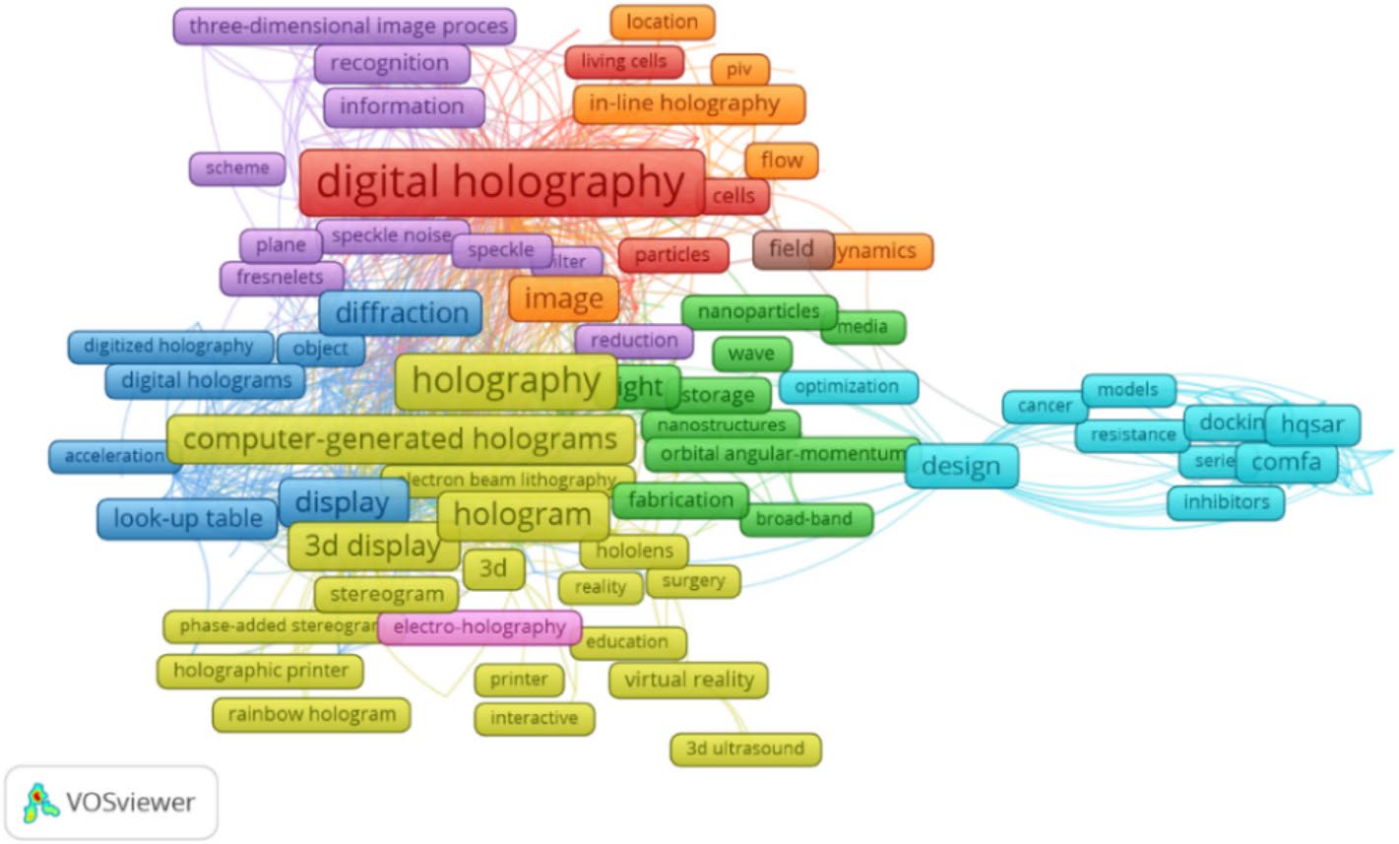
Analysis of keywords in the VOSviewer program (Holo-SDK 1.0 in Unity, https://www.holo-sdk.com/) for the keyword “Hologram 3D”. *Source* own study.

A similar compilation was performed for the keyword Hologram 3D. Approx. 2000 studies were analysed. All of the analysed studies contained 5129 keywords, of which 419 were selected for visualisation. A general map for the keywords is shown in Fig. 8.

In this area, the most common keywords were “digital holography” and “reconstruction” (Table 2).

**Table 2:** The result of keyword search for “Hologram 3D”.

Keyword	Occurrences	Total link strength
Digital holography	349	1271
Reconstruction	223	925
Microscopy	160	656
Holography	183	649
System	122	599
Algorithm	83	405
Phase	84	404
Holographic display	91	400
Computer-generated holograms	94	384
Display	90	373

*Source* own study.

Taking into account the two conducted analyses of scientific articles, keywords related to spatial planning and development as well as real estate appeared in a larger set searched for the keyword “Visualisation 3D” (Table 3). In the second dataset (the keyword searched for: “hologram 3D”), no similar keywords related to the area of spatial planning and development were found.

**Table 3:** The result of keyword search for “Visualisation 3D” in the context of spatial planning and development.

Keyword	Occurrences	Total link strength
Landscape	34	201
Landscape visualisation	21	106
Property	6	23
Planning	23	105

*Source* own study.

The conducted literature review shows that linking a “hologram” to spatial development is still a new issue. Descriptions concerning the use of a LED hologram and pyramid hologram for spatial development presentation are not found in the literature, and the authors believe that they may prove useful for visualising the proposed spatial development. This was confirmed by the feelings of 25 students who were presented the results of visualisation prepared using different 3D visualization techniques and asked to evaluate them. According to them, the greatest value of a hologram is the direct, eyewitness observation that provides an immediate idea of the future condition of the space and the elements of its development. Until now, most people have associated holograms mainly with applications in advertisements and the entertainment industry. The respondents submitted the following opinions and comments:

- The reception of each of the selected technologies varied, yet at the same time, each observer stated clearly that it was a considerably better form of presentation than the classical 2D forms.
- All methods were attractive, with the LED technology-based method being the most highly rated.
- This was a completely new visualisation technique for them, not previously encountered in the areas of spatial planning and development.
- In order to intensify the experience, it was proposed to integrate the image and sound, which, according to the respondents, would further change the level of reception (stronger impact on the observer).

It should be stressed that, despite the unambiguously positive reception of the presented technologies, each of the described and applied methods for “tricking the mind” has its advantages and disadvantages. A holographic pyramid requires no additional equipment from the viewer. The size of the device can be regarded as a disadvantage, since the larger the pyramid, the larger the resulting image. In order to generate a spatial effect, animation in motion should be used, preferably without a background surrounding the object. This significantly limits the presentation of the surrounding space. At the same time, it is important to ensure that the hologram is positioned in a shaded area, as this will result in better reception of the image.

Similarly, when using an LED hologram, the unwanted elements of the background need to be removed. Moreover, to obtain a 3D effect, it is necessary for the object being displayed to be in motion. There are limitations to displaying an object in this case as well. When using a display technology, particular attention should be paid to the occupational health and safety (OHS) rules. The observer needs to maintain a safe distance from the rotating arm, which results in limitations related to the location of such an LED hologram in a room.

The major advantages of anaglyph glasses include a low price (widespread availability) and their compatibility with any display (computer, TV, tablet, smartphone) after the image processing. The disadvantages of this solution include unnatural colours (with red and turquoise colours showing through) and the somewhat unnatural 3D effect. Alternatively, relatively inexpensive passive glasses can be used, but they are also characterised by not very good visual quality. A good, but the most expensive solution, are active glasses offering the possibility of receiving high-resolution images. In addition, these glasses require charging.

Surveys conducted on a group of respondents confirmed that the presented 3D visualisation techniques evoked positive emotions. Despite the above-mentioned limitations, it must be underlined that the 3D visualisation effect evokes positive reactions among viewers and, what is very important, that the hologram technique requires no specialist training or well-developed spatial imagination. This results in a situation where everybody can be an active observer. This technique bridges the gap in imaginative capacity between women and men, between people with specialist training and laypeople, between people using modern technologies and those digitally excluded, etc. In a word, this is a way of 3D visualisation for everybody. It should be noted that the technique is still developing and that spatial planning and development must respond to technological advancements because future adults may not be able to read 2D visualisations. Given that no specialist training is required for this presentation method, it can be a very effective means of visual communication with the public (rationalisation of decisions through an unambiguous reading of the visualisation, as there is no room for interpretation of the presented object).

In conclusion, the use of 3D optical illusion (colloquially called a hologram) in spatial planning and development appears to be justified and is an interesting research trend.

## Data Availability

All data generated or analysed during this study are included in this published article.
